# 1-Hy­droxy-4-methyl­pyridinium chloride

**DOI:** 10.1107/S2414314622010239

**Published:** 2022-10-28

**Authors:** Aarti Saini, Kisturi Dhanwant, Ramalingam Thirumoorthi

**Affiliations:** a Department of Chemistry, School of Chemical Sciences and Pharmacy, Central, University of Rajasthan, NH8, Bandarsindri, Ajmer-305817, India; University of Aberdeen, United Kingdom

**Keywords:** chloride ion, crystal structure, hydrogen bond, hy­droxy group, pyridinium ion

## Abstract

The title salt, C_6_H_8_NO^+^·Cl^−^, contains two cations and two anions in the asymmetric unit. The components are linked by O—H⋯Cl and C—H⋯Cl hydrogen bonds, leading to tetra- (square-planar) or penta-coordinated (square-pyramidal) chloride ions. The title salt is isostructural with its bromide analogue.

## Abstract 2

The title salt contains two cations and two anions in the asymmetric unit.

## Graphical abstract

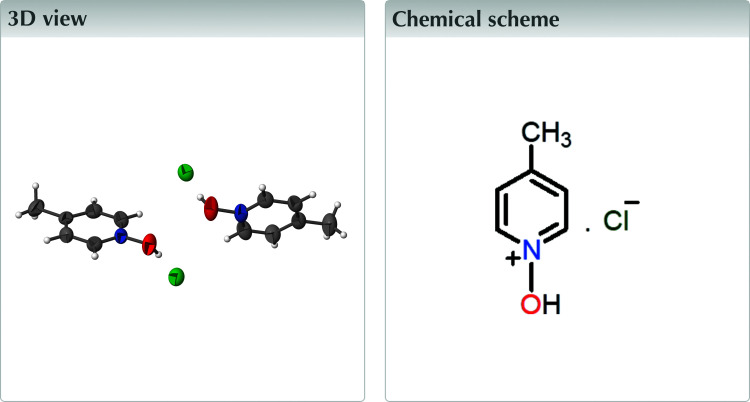

## Structure description

**Figure 1:**
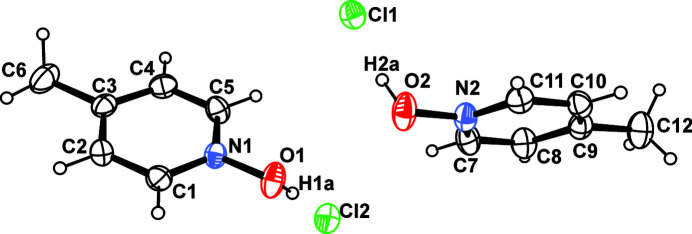
*ORTEP* diagram of **1** with 50% displacement ellipsoid probability level.

The title mol­ecular salt, C_6_H_8_NO^+^·Cl^−^, **1**, crystallizes with two 1-hy­droxy-4-methyl­pyridinium cations and two chloride anions in the asymmetric unit in space group *P*2_1_/*c*, indicating that proton transfer has occurred from HCl to the *N*-oxide O atoms (Fig. 1 ▸). The N—O bond lengths are 1.371 (2) and 1.379 (2) Å, which are comparable to its bromide analogue **2** (1.373 and 1.374 Å; Ryzhakova *et al.*, 2012 ▸). The average N—O—H bond angle in **1** [103.9 (19)°] is significantly smaller compared to **2** (110.9°). However, the torsion angles C_Ar_—N—O—H in **1** (62.9 and 57.4°) are very similar to those in **2** (62.8 and 57.6°).

**Figure 2:**
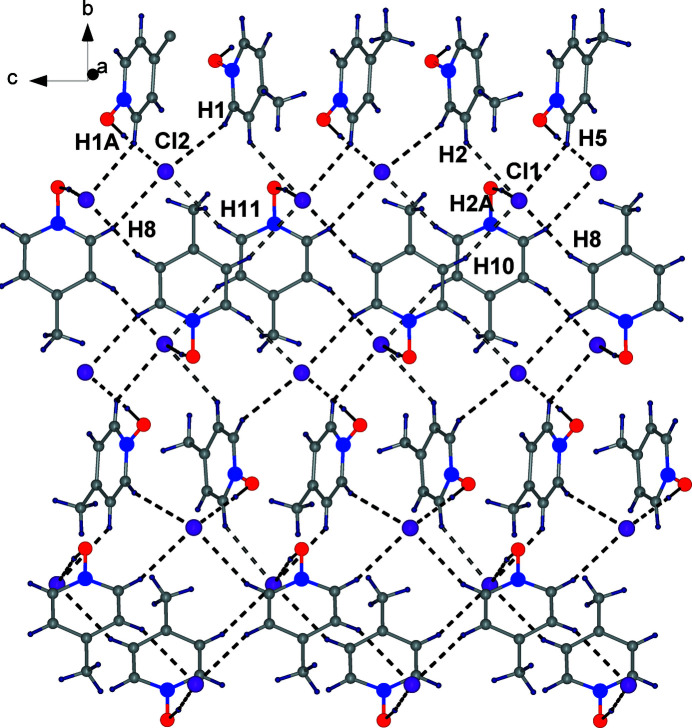
Two-dimensional packing diagram of **1**.

In the extended structure of **1**, one of the cations provides four hydrogen-bond donors (three C—H groupings and one O—H group) while the other cation provides five hydrogen-bond donors, *i.e.*, one from the O—H group and four from C—H centres, all with chloride ion acceptors to form tetra­/penta-coordinated anions (Table 1 ▸; Fig. 2 ▸). As expected, the H⋯Cl separation for the O—H⋯Cl hydrogen bonds (mean 1.97 Å) is much shorter than the H⋯Cl separation for the C—H⋯Cl hydrogen bonds (mean 2.79 Å).

## Synthesis and crystallization

**Figure 3:**
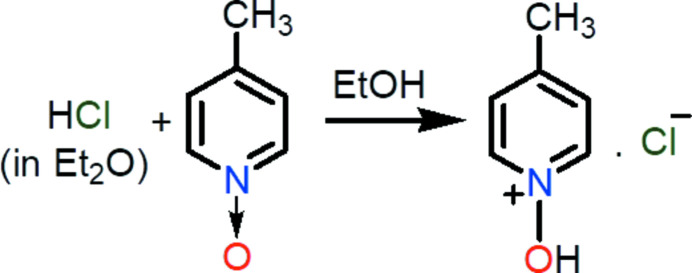
Reaction scheme.

A 2.0 *M* solution of hydro­chloric acid in Et_2_O (0.167 ml, 5.49 mmol) was added dropwise to an ethanol (3 ml) solution of 4-methyl­pyridine *N*-oxide (0.2 g, 1.83 mmol). The reaction scheme is shown in Fig. 3 ▸. The reaction mixture was stirred for 2 h at room temperature followed by solvent evaporation using a rotary evaporator to obtain a solid. The obtained product was washed with diethyl ether and dried to get colorless solid. Yield: 82%, m.p. 114°C. ^1^H NMR (500 MHz, CDCl_3_, p.p.m.) δ 8.81 (*d*, 2H, *J* = 6.5 Hz), 7.70 (*d*, 2H, *J* = 6.5 Hz), 2.62 (*s*, 3H). ^1^H NMR (CD_3_OD, 500 MHz, p.p.m.): δ 8.70 (*d*, 2H, *J* = 6.5 Hz), 7.96 (*d*, 2H, *J* = 6 Hz), 2.70 (*s*, 3H). ^13^C{^1^H} NMR (CDCl_3_, 125 MHz, p.p.m.): 154.0, 138.9, 128.5, 21.7.

The slow evaporation of a di­chloro­methane solution of **1** produced good quality, pale-yellow, rhombus-shaped crystals.

## Refinement

Crystal data, data collection and structure refinement details are summarized in Table 2 ▸.

## Supplementary Material

Crystal structure: contains datablock(s) I. DOI: 10.1107/S2414314622010239/hb4415sup1.cif

Structure factors: contains datablock(s) I. DOI: 10.1107/S2414314622010239/hb4415Isup2.hkl

Supporting information file. DOI: 10.1107/S2414314622010239/hb4415Isup3.cml

CCDC reference: 2215272

Additional supporting information:  crystallographic information; 3D view; checkCIF report

## Figures and Tables

**Table 1:** Hydrogen-bond geometry (Å, °)

*D*—H⋯*A*	*D*—H	H⋯*A*	*D*⋯*A*	*D*—H⋯*A*
O1—H1*A*⋯Cl2	0.86 (3)	2.03 (3)	2.8847 (19)	175 (3)
O2—H2*A*⋯Cl1	0.98 (3)	1.91 (3)	2.879 (2)	172 (3)
C2—H2⋯Cl1^i^	0.93	2.81	3.567 (2)	139
C5—H5⋯Cl1	0.93	2.73	3.519 (2)	143
C7—H7⋯Cl2	0.93	2.79	3.652 (3)	154
C8—H8⋯Cl1^ii^	0.93	2.72	3.618 (3)	162
C10—H10⋯Cl1^iii^	0.93	2.81	3.672 (2)	155
C11—H11⋯Cl2^iv^	0.93	2.74	3.646 (2)	164

Symmetry codes: (i) 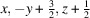 ; (ii) 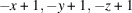 ; (iii) 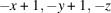 ; (iv) 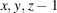 .

**Table 2:** Experimental details

Crystal data	
Chemical formula	C_6_H_8_NO^+^·Cl^−^
*M*_r_	145.58
Crystal system, space group	Monoclinic, *P*2_1_/*c*
Temperature (K)	296
*a*, *b*, *c* (Å)	7.1610 (16), 26.271 (6), 7.7474 (17)
β (°)	95.495 (3)
*V* (Å^3^)	1450.8 (6)
*Z*	8
Radiation type	Mo *K*α
μ (mm^−1^)	0.44
Crystal size (mm)	0.11 × 0.09 × 0.06

Data collection	
Diffractometer	Bruker APEXII CCD
Absorption correction	Multi-scan (*SADABS*; Bruker, 2014 ▸)
*T*_min_, *T*_max_	0.953, 0.974
No. of measured, independent and observed [*I* > 2σ(*I*)] reflections	41123, 3354, 2663
*R*_int_	0.051
(sin θ/λ)_max_ (Å^−1^)	0.652

Refinement	
*R*[*F* ^2^ > 2σ(*F* ^2^)], *wR*(*F* ^2^), *S*	0.042, 0.129, 1.06
No. of reflections	3354
No. of parameters	171
H-atom treatment	H atoms treated by a mixture of independent and constrained refinement
Δρ_max_, Δρ_min_ (e Å^−3^)	0.34, −0.26

Computer programs: *APEX2* (Bruker, 2014 ▸), *SAINT* (Bruker, 2013 ▸), *SHELXS97* (Sheldrick, 2008 ▸), *SHELXL* (Sheldrick, 2015 ▸), *ORTEP-3 for Windows* (Farrugia, 2012 ▸) and *publCIF* (Westrip, 2010 ▸).

## full crystallographic data

### Crystal data

**Table:**

C_6_H_8_NO^+^·Cl^−^	*F*(000) = 608
*M**_r_* = 145.58	*D*_x_ = 1.333 Mg m^−^^3^
Monoclinic, *P*2_1_/*c*	Mo *K*α radiation, λ = 0.71073 Å
*a* = 7.1610 (16) Å	Cell parameters from 9290 reflections
*b* = 26.271 (6) Å	θ = 2.8–24.5°
*c* = 7.7474 (17) Å	µ = 0.44 mm^−^^1^
β = 95.495 (3)°	*T* = 296 K
*V* = 1450.8 (6) Å^3^	Needle, colorless
*Z* = 8	0.11 × 0.09 × 0.06 mm

### Data collection

**Table:**

Bruker APEXII CCD diffractometer	2663 reflections with *I* > 2σ(*I*)
ω scans	*R*_int_ = 0.051
Absorption correction: multi-scan (SADABS; Bruker, 2014)	θ_max_ = 27.6°, θ_min_ = 1.6°
*T*_min_ = 0.953, *T*_max_ = 0.974	*h* = −9→9
41123 measured reflections	*k* = −34→34
3354 independent reflections	*l* = −10→10

### Refinement

**Table:**

Refinement on *F*^2^	Primary atom site location: structure-invariant direct methods
Least-squares matrix: full	Hydrogen site location: mixed
*R*[*F*^2^ > 2σ(*F*^2^)] = 0.042	H atoms treated by a mixture of independent and constrained refinement
*wR*(*F*^2^) = 0.129	*w* = 1/[σ^2^(*F*_o_^2^) + (0.0646*P*)^2^ + 0.4782*P*] where *P* = (*F*_o_^2^ + 2*F*_c_^2^)/3
*S* = 1.06	(Δ/σ)_max_ = 0.001
3354 reflections	Δρ_max_ = 0.34 e Å^−^^3^
171 parameters	Δρ_min_ = −0.25 e Å^−^^3^
0 restraints	

### Special details

**Table:**

Geometry. All esds (except the esd in the dihedral angle between two l.s. planes) are estimated using the full covariance matrix. The cell esds are taken into account individually in the estimation of esds in distances, angles and torsion angles; correlations between esds in cell parameters are only used when they are defined by crystal symmetry. An approximate (isotropic) treatment of cell esds is used for estimating esds involving l.s. planes.

### Fractional atomic coordinates and isotropic or equivalent isotropic displacement parameters (Å^2^)

**Table:**

	*x*	*y*	*z*	*U*_iso_*/*U*_eq_	
C1	0.3022 (3)	0.78000 (7)	0.4800 (3)	0.0457 (4)	
H1	0.400319	0.800303	0.449620	0.055*	
C2	0.1436 (3)	0.80150 (8)	0.5313 (3)	0.0500 (5)	
H2	0.133207	0.836765	0.534577	0.060*	
C3	−0.0028 (3)	0.77163 (8)	0.5785 (2)	0.0448 (4)	
C4	0.0191 (3)	0.71936 (8)	0.5702 (3)	0.0496 (5)	
H4	−0.075402	0.698063	0.602249	0.060*	
C5	0.1785 (3)	0.69878 (8)	0.5152 (3)	0.0488 (5)	
H5	0.191540	0.663677	0.506589	0.059*	
C6	−0.1788 (3)	0.79546 (12)	0.6313 (3)	0.0711 (7)	
H6A	−0.166953	0.831849	0.629104	0.107*	
H6B	−0.283357	0.785194	0.552116	0.107*	
H6C	−0.198791	0.784606	0.746382	0.107*	
C7	0.6459 (3)	0.54962 (9)	0.3558 (3)	0.0571 (5)	
H7	0.608047	0.564925	0.454901	0.069*	
C8	0.7375 (3)	0.50418 (9)	0.3661 (3)	0.0564 (5)	
H8	0.761757	0.488208	0.472987	0.068*	
C9	0.7952 (3)	0.48143 (8)	0.2181 (3)	0.0485 (5)	
C10	0.7595 (3)	0.50710 (8)	0.0630 (3)	0.0477 (5)	
H10	0.799599	0.493306	−0.037661	0.057*	
C11	0.6660 (3)	0.55255 (8)	0.0555 (3)	0.0477 (5)	
H11	0.641024	0.569680	−0.049225	0.057*	
C12	0.8943 (4)	0.43125 (10)	0.2289 (4)	0.0741 (7)	
H12A	0.905880	0.419521	0.346807	0.111*	
H12B	0.823683	0.406943	0.156757	0.111*	
H12C	1.016852	0.435067	0.190073	0.111*	
N1	0.3148 (2)	0.72972 (6)	0.47417 (19)	0.0405 (4)	
N2	0.6116 (2)	0.57173 (6)	0.2021 (2)	0.0474 (4)	
CL1	0.15453 (8)	0.58405 (2)	0.27982 (7)	0.05342 (17)	
CL2	0.59963 (8)	0.64306 (2)	0.69818 (7)	0.05963 (19)	
O1	0.4730 (2)	0.70951 (7)	0.4144 (2)	0.0599 (4)	
O2	0.5160 (3)	0.61737 (6)	0.1905 (3)	0.0670 (5)	
H1A	0.517 (4)	0.6892 (11)	0.495 (4)	0.084 (10)*	
H2A	0.393 (4)	0.6090 (12)	0.228 (4)	0.088 (9)*	

### Atomic displacement parameters (Å^2^)

**Table:**

	*U*^11^	*U*^22^	*U*^33^	*U*^12^	*U*^13^	*U*^23^
C1	0.0450 (10)	0.0393 (10)	0.0525 (11)	−0.0037 (8)	0.0041 (8)	0.0030 (8)
C2	0.0510 (11)	0.0393 (10)	0.0586 (12)	0.0063 (8)	0.0000 (9)	−0.0050 (9)
C3	0.0394 (9)	0.0594 (12)	0.0348 (9)	0.0058 (8)	−0.0009 (7)	−0.0047 (8)
C4	0.0439 (10)	0.0559 (12)	0.0493 (11)	−0.0069 (9)	0.0056 (8)	0.0053 (9)
C5	0.0588 (12)	0.0363 (10)	0.0524 (11)	−0.0026 (8)	0.0114 (9)	0.0003 (8)
C6	0.0472 (12)	0.099 (2)	0.0664 (15)	0.0218 (12)	0.0036 (11)	−0.0100 (14)
C7	0.0726 (14)	0.0504 (12)	0.0525 (12)	0.0004 (10)	0.0271 (10)	−0.0030 (10)
C8	0.0690 (14)	0.0532 (12)	0.0494 (12)	0.0051 (10)	0.0174 (10)	0.0066 (10)
C9	0.0457 (10)	0.0430 (10)	0.0585 (12)	−0.0016 (8)	0.0146 (9)	0.0006 (9)
C10	0.0516 (11)	0.0458 (11)	0.0476 (11)	−0.0038 (9)	0.0145 (9)	−0.0070 (9)
C11	0.0477 (10)	0.0474 (11)	0.0492 (11)	−0.0048 (8)	0.0116 (8)	0.0034 (9)
C12	0.0871 (19)	0.0566 (14)	0.0821 (18)	0.0209 (13)	0.0252 (14)	0.0068 (13)
N1	0.0429 (8)	0.0426 (8)	0.0373 (8)	0.0066 (7)	0.0097 (6)	−0.0004 (6)
N2	0.0494 (9)	0.0346 (8)	0.0616 (10)	0.0001 (7)	0.0216 (8)	0.0019 (7)
CL1	0.0606 (3)	0.0456 (3)	0.0555 (3)	−0.0034 (2)	0.0130 (2)	0.0013 (2)
CL2	0.0654 (4)	0.0611 (4)	0.0534 (3)	0.0105 (3)	0.0104 (2)	0.0046 (2)
O1	0.0613 (9)	0.0643 (10)	0.0585 (9)	0.0200 (8)	0.0288 (8)	0.0073 (8)
O2	0.0717 (11)	0.0387 (8)	0.0967 (13)	0.0096 (7)	0.0401 (9)	0.0097 (8)

### Geometric parameters (Å, º)

**Table:**

C1—N1	1.325 (3)	C7—H7	0.9300
C1—C2	1.361 (3)	C8—C9	1.390 (3)
C1—H1	0.9300	C8—H8	0.9300
C2—C3	1.386 (3)	C9—C10	1.380 (3)
C2—H2	0.9300	C9—C12	1.495 (3)
C3—C4	1.384 (3)	C10—C11	1.367 (3)
C3—C6	1.499 (3)	C10—H10	0.9300
C4—C5	1.367 (3)	C11—N2	1.335 (3)
C4—H4	0.9300	C11—H11	0.9300
C5—N1	1.332 (3)	C12—H12A	0.9600
C5—H5	0.9300	C12—H12B	0.9600
C6—H6A	0.9600	C12—H12C	0.9600
C6—H6B	0.9600	N1—O1	1.371 (2)
C6—H6C	0.9600	N2—O2	1.379 (2)
C7—N2	1.326 (3)	O1—H1A	0.86 (3)
C7—C8	1.361 (3)	O2—H2A	0.98 (3)
			
N1—C1—C2	118.98 (19)	C7—C8—H8	119.7
N1—C1—H1	120.5	C9—C8—H8	119.7
C2—C1—H1	120.5	C10—C9—C8	117.57 (19)
C1—C2—C3	121.03 (19)	C10—C9—C12	121.8 (2)
C1—C2—H2	119.5	C8—C9—C12	120.7 (2)
C3—C2—H2	119.5	C11—C10—C9	120.85 (19)
C4—C3—C2	117.21 (18)	C11—C10—H10	119.6
C4—C3—C6	122.0 (2)	C9—C10—H10	119.6
C2—C3—C6	120.8 (2)	N2—C11—C10	118.36 (19)
C5—C4—C3	120.56 (19)	N2—C11—H11	120.8
C5—C4—H4	119.7	C10—C11—H11	120.8
C3—C4—H4	119.7	C9—C12—H12A	109.5
N1—C5—C4	119.06 (19)	C9—C12—H12B	109.5
N1—C5—H5	120.5	H12A—C12—H12B	109.5
C4—C5—H5	120.5	C9—C12—H12C	109.5
C3—C6—H6A	109.5	H12A—C12—H12C	109.5
C3—C6—H6B	109.5	H12B—C12—H12C	109.5
H6A—C6—H6B	109.5	C1—N1—C5	123.14 (17)
C3—C6—H6C	109.5	C1—N1—O1	117.29 (16)
H6A—C6—H6C	109.5	C5—N1—O1	119.49 (16)
H6B—C6—H6C	109.5	C7—N2—C11	123.68 (18)
N2—C7—C8	118.9 (2)	C7—N2—O2	119.18 (17)
N2—C7—H7	120.5	C11—N2—O2	117.14 (18)
C8—C7—H7	120.5	N1—O1—H1A	104 (2)
C7—C8—C9	120.6 (2)	N2—O2—H2A	103.9 (18)
			
N1—C1—C2—C3	0.6 (3)	C12—C9—C10—C11	178.9 (2)
C1—C2—C3—C4	−0.4 (3)	C9—C10—C11—N2	0.6 (3)
C1—C2—C3—C6	−178.7 (2)	C2—C1—N1—C5	0.4 (3)
C2—C3—C4—C5	−0.8 (3)	C2—C1—N1—O1	177.09 (18)
C6—C3—C4—C5	177.5 (2)	C4—C5—N1—C1	−1.6 (3)
C3—C4—C5—N1	1.8 (3)	C4—C5—N1—O1	−178.24 (18)
N2—C7—C8—C9	0.4 (4)	C8—C7—N2—C11	−1.7 (3)
C7—C8—C9—C10	1.3 (3)	C8—C7—N2—O2	178.9 (2)
C7—C8—C9—C12	−179.4 (2)	C10—C11—N2—C7	1.2 (3)
C8—C9—C10—C11	−1.8 (3)	C10—C11—N2—O2	−179.39 (18)

### Hydrogen-bond geometry (Å, º)

**Table:**

*D*—H···*A*	*D*—H	H···*A*	*D*···*A*	*D*—H···*A*
O1—H1*A*···Cl2	0.86 (3)	2.03 (3)	2.8847 (19)	175 (3)
O2—H2*A*···Cl1	0.98 (3)	1.91 (3)	2.879 (2)	172 (3)
C2—H2···Cl1^i^	0.93	2.81	3.567 (2)	139
C5—H5···Cl1	0.93	2.73	3.519 (2)	143
C7—H7···Cl2	0.93	2.79	3.652 (3)	154
C8—H8···Cl1^ii^	0.93	2.72	3.618 (3)	162
C10—H10···Cl1^iii^	0.93	2.81	3.672 (2)	155
C11—H11···Cl2^iv^	0.93	2.74	3.646 (2)	164

Symmetry codes: (i) *x*, −*y*+3/2, *z*+1/2; (ii) −*x*+1, −*y*+1, −*z*+1; (iii) −*x*+1, −*y*+1, −*z*; (iv) *x*, *y*, *z*−1.
